# Pathways to win-wins or trade-offs? How certified community forests impact forest restoration and human wellbeing

**DOI:** 10.1098/rstb.2021.0080

**Published:** 2023-01-02

**Authors:** Robin Loveridge, Andrew R. Marshall, Marion Pfeifer, Steven Rushton, Petro P. Nnyiti, Lilian Fredy, Susannah M. Sallu

**Affiliations:** ^1^ Department of Environment and Geography, University of York, York, YO10 5NG, UK; ^2^ The Biodiversity Consultancy, Cambridge, CB2 1SJ, UK; ^3^ Forest Research Institute, University of the Sunshine Coast, QLD 4655, Australia; ^4^ Flamingo Land Ltd, Kirby Misperton, North Yorkshire, YO17 6UX, UK; ^5^ School of Natural and Environmental Sciences, TROPS lab, Newcastle University, Newcastle upon Tyne, NE1 7RU, UK; ^6^ Institute of Adult Education, Dar es Salaam, 57QM+4JF, Tanzania; ^7^ Sokoine University of Agriculture, Morogoro, 4MX5+52, Tanzania; ^8^ School of Earth and Environment, University of Leeds, Leeds, LS2 9JT, UK

**Keywords:** pathways, protected areas, forest certification, restoration, community forests, wellbeing

## Abstract

Certified community forests combine local governance with forest certification and aim to serve multiple objectives including forest protection, restoration, human wellbeing and equitable governance. However, the causal pathways by which they impact these objectives remain poorly understood. The ability of protected area impact evaluations to identify complex pathways is limited by a narrow focus on top-down theoretical, quantitative perspectives and inadequate consideration of local context. We used a novel mixed-methods research design that integrates the perspectives of multiple actors to develop a generalized conceptual model of the causal pathways for certified community forests. We tested the model using a combination of statistical matching, structural equation modelling and qualitative analyses for an agroforestry landscape in Tanzania. We found certified community forests positively impacted human wellbeing, equitable governance and forest restoration. Equitable governance had the largest impact on wellbeing, followed by crop yield and forest resource availability. Timber revenues varied widely between villages and the average effect of financial benefits did not impact wellbeing due to the immature stage of the certified timber market. We identified positive interactions and trade-offs between conservation and agriculture. Our findings suggest that no simple solution exists for meeting multiple objectives. However, developing understanding of the pathways linking social and conservation outcomes can help identify opportunities to promote synergies and mitigate negative impacts to reconcile competing objectives.

This article is part of the theme issue ‘Understanding forest landscape restoration: reinforcing scientific foundations for the UN Decade on Ecosystem Restoration’.

## 1. Introduction

Protected areas (PAs) have traditionally been established in areas of biodiversity importance with the objectives of protecting and restoring nature. However, over the last 50 years the roles of PAs have expanded to include human wellbeing and equitable governance objectives [1,2]. Some contend that these objectives are mutually supporting, with ‘win-win’ outcomes possible for people and nature [3]. However, others consider these objectives to be competing [1]. For example, farming is the dominant livelihood of rural communities in low-income countries and a key determinant of human wellbeing [4]. While agricultural expansion is also the primary driver of forest loss in the tropics and a major driver of degradation within PAs [5]. To date, trade-offs between objectives are more common than win-wins [6]. Therefore, there is a need to re-evaluate the core assumptions underpinning win-win framings of PA governance [7], to understand if and how these multiple objectives can be reconciled.

Certified Community Forests (CFs) represent a new generation of PAs seeking to meet the expanding role of PAs, by combining two recent trends in forest PA governance: (i) decentralization—transferring governance responsibility from central governments to local actors in efforts to enhance equitable governance; (ii) forest certification to increase the financial benefits and equitable benefit sharing to incentivize sustainable management [8]. Forest landscape restoration and other conservation approaches have also undergone parallel evolutions in governance to integrate social objectives. These new trends create additional complexity as international forest certification requirements are superimposed on local governance arrangements [9]. Furthermore, diverse forms of certified CFs have evolved as governance arrangements are adapted to align with local cultural norms in efforts to create forms of governance that are considered locally legitimate [10]. Perceptions of fair and equitable governance are increasingly associated with positive wellbeing outcomes and effective conservation [11]. To understand and improve effective PA governance to serve multiple objectives, greater understanding is needed of how complex PA governance arrangements impact multiple objectives [12], and the local contextual factors that determine success and failure [13]. Yet few rigorous impact evaluations of certified CFs exist [14].

**Figure 1.**
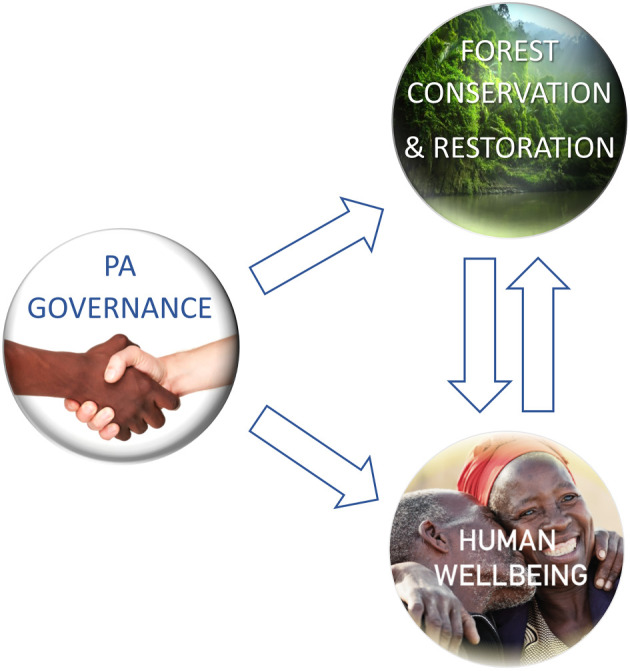
Conceptual model of generalized pathways between protected area (PA) governance, forests and human wellbeing. (Online version in colour.)

Analysis of causal pathways can be used to explain the complex processes by which PAs impact outcomes [7]. Win-win outcomes require that forest PA governance leads to simultaneously positive impacts on forests and human wellbeing (figure 1). Furthermore, win-win framings depend on the core assumption that human wellbeing is strongly linked to the natural environment [15]. Therefore, good environmental governance will benefit people, with positive interacting pathways between social and ecological outcomes (figure 1). Specific interaction pathways might include (i) forests to people—effective PA governance increasing the abundance of those forest products and ecosystem benefits (monetary and non-monetary) used by adjacent communities, resulting in improvements in human wellbeing, (ii) people to forests—positive attitudes towards conservation result in improved conservation outcomes [6]. These interaction pathways influence how people perceive and respond to change [16] and may positively reinforce positive outcomes [17].

However, the presence of negative interactions between social and ecological outcomes would result in trade-offs between PA objectives. For example, if conservation governance is perceived as being unfair, this could lead to conflicts and local resistance [18], as occurred in Kilimanjaro, Tanzania, where retaliatory killings of elephants have been recorded as a form of protest against PA conservation [19]. The presence of negative interaction pathways would constrain the ability of conservation interventions to achieve win-wins.

Advances in the field of impact evaluation have seen the mainstreaming of statistical matching to exclude alternative explanations and attribute observed differences to the intervention [20]. However, this approach has limited ability to assess the pathways by which PAs cause impacts because of a focus on (i) ends, rather than means, (ii) a narrow suite of pre-determined hypotheses, rather than open-ended consideration of multiple contextual drivers. Firstly, evaluations tend to use single response variables to focus on long-term impacts [13], but the mechanisms through which these impacts are achieved remain largely untested, though for an exception see [7]. For example, improving equitable resource governance is one mechanism by which PAs can improve human wellbeing [21]; however, the relationship between equitable governance and wellbeing is rarely assessed [22]. Use of a conceptual model provides a powerful alternative approach for unpacking complex impacts into causally linked short- and long-term outcomes [23].

Secondly, impact evaluations tend to test *a priori* hypotheses of how PAs impact wellbeing or conservation using exclusively quantitative approaches. This top-down framing of a study system excludes local perspectives and has been described as creating a ‘bottleneck’ in dialogue [24], which may miss locally relevant, unanticipated and alternative explanations for PA impacts [25,26]. Greater integration of the perspectives of local actors in the design of impact evaluations has potential to provide more comprehensive understanding of governance challenges and provide novel insights.

We aim to advance methods to evaluate the success of conservation and restoration interventions. We identify and test the causal pathways by which certified CFs impact human wellbeing and forest restoration for a case study in Tanzania. Specifically, we evaluate win-win assumptions of PA governance by testing pathways of (i) equitable governance, (ii) financial benefits, (iii) interaction effects and (iv) trade-offs.

We advance on existing evaluation methodologies by (i) combining statistical matching with a conceptual model (ii) integrating top-down theoretical perspectives with bottom-up perspectives of local actors to promote inclusive consideration of alternative explanations from marginalized actors. We thereby contribute to two key knowledge gaps of the United Nations Decade on Restoration: (i) methods for designing interventions, and monitoring restoration success; (ii) linkages between the health of ecosystems and the flow of services to communities [27].

## 2. Methodology

### (a) Study site and sampling

**Figure 2.**
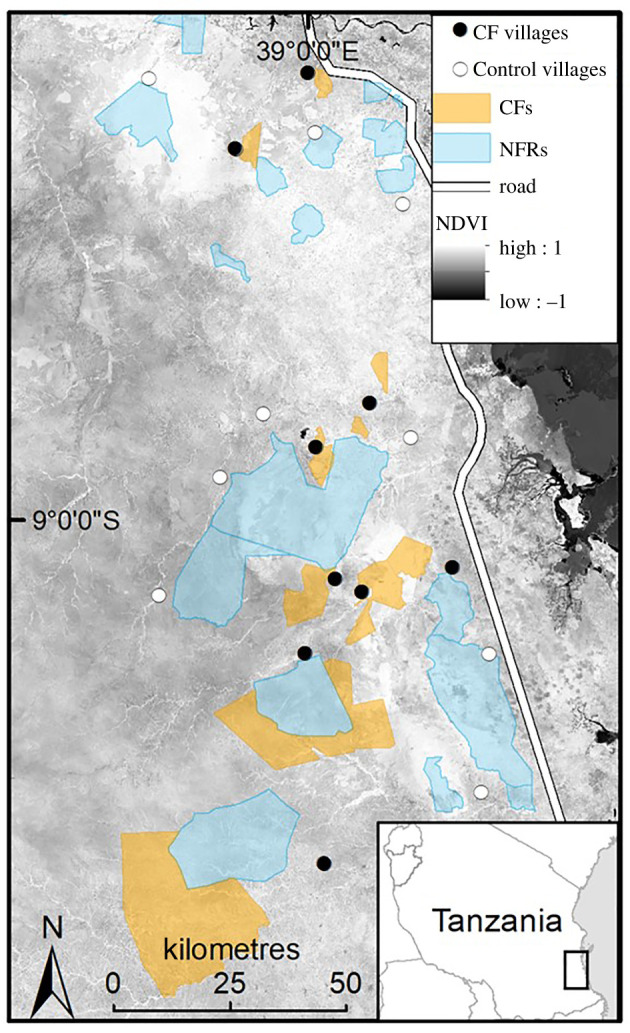
Study landscape in Lindi and Pwani regions of Tanzania detailing the nine certified CF villages and adjacent National forest Reserves (NFRs) and 10 control villages. (Online version in colour.)

Tanzania provides an excellent test case of the challenges to reconcile forest restoration and human wellbeing objectives as national development policies aim to expand both agricultural and PA land uses, while CFs are often established on forests already considered to be degraded and economically marginal [28]. We focus on a PA-dominated landscape in Eastern Tanzania, where certified CFs have an established history and community governance is supported by district government and an NGO, Mpingo Conservation & Development Initiative (MCDI) (figure 2).

### (b) Developing a conceptual model

**Figure 3.**
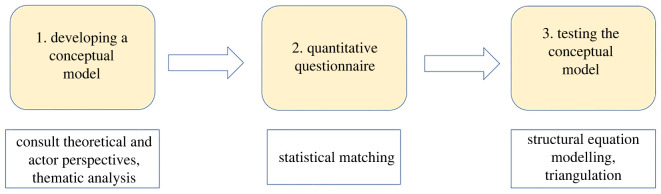
Steps (above) and methods (below) used to develop and test the conceptual model. (Online version in colour.)

Our conceptual model aimed to integrate actor perspectives with hypothesized pathways derived from conservation science literature (figure 3). We consulted national, regional and local actors involved in forest PAs and forest certification, in total undertaking 30 focus groups and 34 key informant interviews between 2018 and 2019 (electronic supplementary material). Questions concerned how PAs influenced conservation and human wellbeing and PA governance processes and challenges.

A thematic analysis of transcripts was then undertaken to identify actor perspectives of the main pathways linking certified CFs with conservation and human wellbeing impacts by identifying logical causal statements that were similar between independent consultation sessions. Identified pathways were then converted into a connected sequence of indicators linking CF governance via one or more causally linked mechanisms to human wellbeing and conservation impacts (electronic supplementary material). The overall conceptual model was then composed of these main pathways. Indicators may be connected to more than one pathway if the causal logic suggested interactions between indicators from different pathways.

All indicators in the conceptual model were included in a quantitative questionnaire to collect data on community perceptions of all indicators, which were then used to test the model. Our conceptual model emphasizes measuring ‘bottom-up’ community perceptions, rather than externally measured data sources because perceptions are important drivers of local behaviour and success of conservation interventions [29].

### (c) Matched sample selection

To improve the causal inference of our study we used statistical matching to compare certified CFs to control villages that represent the counterfactual situation—what would have happened in the absence of the intervention [30]. This approach helps to exclude alternative explanations and attribute observed differences to the PA [20]. We, therefore, define impact as the difference in response indicators between the sampled certified CF villages and matched control villages. We defined treatment as certified CFs that have been established and participated in forest certification for at least 5 years. Therefore, all certified CFs had completed at least one 5-year management cycle of harvesting, revenue disbursement and external assessment by an FSC auditor, providing assurance that the required governance processes were being implemented and the villages represented ‘de facto’ examples of this governance approach. All nine villages that met these criteria were matched to 10 control villages using nearest neighbour matching implemented in the ‘Matchit’ package in R v. 3.5.3 to select treatment and control villages with balanced distributions of a suite of confounding socio-environmental variables (electronic supplementary material). The statistically selected control villages were then reviewed by an expert panel of national actors to confirm the appropriateness of empirically determined matches from the perspective of national actors (electronic supplementary material).

The questionnaire was undertaken with 955 people from the nine villages with certified CFs and 10 matched control villages, with at least 50 respondents per village stratified by gender, local elite status (village government representative, 0/1) and wealth category (electronic supplementary material). The questionnaire was undertaken at the scale of individuals rather than whole households since the concept of wellbeing contains subjective elements which cannot be generalized across households [23].

### (d) Response indicators

#### (i) Human wellbeing

Multidimensional human wellbeing provides a comprehensive measure of social impacts. Wellbeing indicators were identified following the Wellbeing Indicator Selection Protocol [31] by a subset of eight focus groups with separate groups of women, men, elites and non-elites in target communities. During the focus groups, locally relevant indicators were agreed for five broad domains of wellbeing; (i) material wellbeing, (ii) health, (iii) social relations, (iv) security, (v) freedom of choice and action [32]. Through stepwise reduction of questionnaire data, we produced a final list of 25 orthogonal indicators representative of the five domains of wellbeing. A Human Wellbeing Index (HWI) was then calculated as the mean score of each domain mean to provide a single response indicator (electronic supplementary material).

#### (ii) Forest restoration

Miombo woodlands were the dominant forest type in the study landscape. Normalised difference vegetation index (NDVI) correlates with ground vegetation biomass and productivity under low to medium vegetation density conditions such as the Miombo woodlands [33]. NDVI change, therefore, provides an appropriate proxy measure of forest recovery. We calculated NDVI change between 2014 and 2019 as a proxy measure of conservation effectiveness on village land (electronic supplementary material). NDVI change was calculated for each survey respondent as the mean of 30 × 30 m pixels within a 10 km radius of the respondent's house as determined by GPS during interview. In this way the conservation effectiveness indicator concerns sustainable forest management on village land surrounding the respondent's home.

#### (iii) Equitable governance

Equitable governance concerns notions of fairness [2], in relation to three dimensions: (i) distribution—the fair distribution of benefits and costs; (ii) recognition—respect for the rights and values of different actors; (iii) procedure—the fair participation of actors in decision-making, relating to transparency of information, accountability of managers and equitable dispute resolution. Guided by the IUCN and IIED good PA governance assessment methodologies [34–36], we included questions on governance challenges in exploratory consultations. Based on actor perspectives of governance challenges we included locally relevant equitable governance indicators of all three dimensions of equitable governance in the quantitative questionnaire (electronic supplementary material). An overall governance equity index was generated by confirmatory factor analysis (CFA) of candidate governance indicators from the three dimensions of governance equity.

### (e) Testing the conceptual model

We tested the conceptual model by structural equation modelling using PiecewiseSEM in R [37]. Latent variables were first modelled by confirmatory factor analysis in the Lavaan package in R [38], then the latent variable constructs were passed into the PiecewiseSEM package for modelling. This approach is justified because PiecewiseSEM does not use a global covariance matrix and so the covariance components for the individual latent variables are not required for solution of the overall path model. Furthermore, we were particularly interested in adjusting for variation due to village to make the analysis more generic and latent variables are not easily derived in a mixed effect framework. We then fitted (generalized) linear mixed effects models using PiecewiseSEM to account for the hierarchal data structure of interview respondents being nested within villages. For binary response variables, we used binomial error distribution in generalized linear mixed effects models.

We included socio-economic indicators for local elite status and gender in models to account for any systematic perception biases between actors. To account for residual imbalances in the distributions of confounding variables between treatment and control groups we included orthogonal sets of confounding variables as predictors of response variables [39]. We then performed model simplification to achieve statistical parsimony i.e. the minimum complexity necessary to describe key relationships [40]. For each linear mixed effects model in turn, we sequentially removed the least explanatory predictor variable from the full model if its deletion caused a reduction in Akaike's information criterion.

For continuous variables, we report standardized path coefficients, which estimate the expected change in the response variable (e.g. wellbeing) as a function of the change in the explanatory variable (e.g. equitable governance), in units of standard deviation. For categorical variables (e.g. governance treatment versus control group), we report the model-estimated means for each factor level [37]. For all response variables we report the marginal *r*^2^ values (the variance explained by fixed effects).

We assessed the overall model fit by Shipley's test of d-separation, accepted when Fisher's C statistic is higher than a significance level (*p* < 0.05). Finally, we critically reviewed the conceptual model through triangulation with the qualitative data to assess whether the identified pathways and trends were representative of different village cases and actors sampled. This served as a verification check to ensure inclusive representation of pluralistic perspectives, particularly potentially marginalized or minority actors whose perspectives might otherwise be masked by reporting normative trends.

## 3. Results

**Figure 4.**
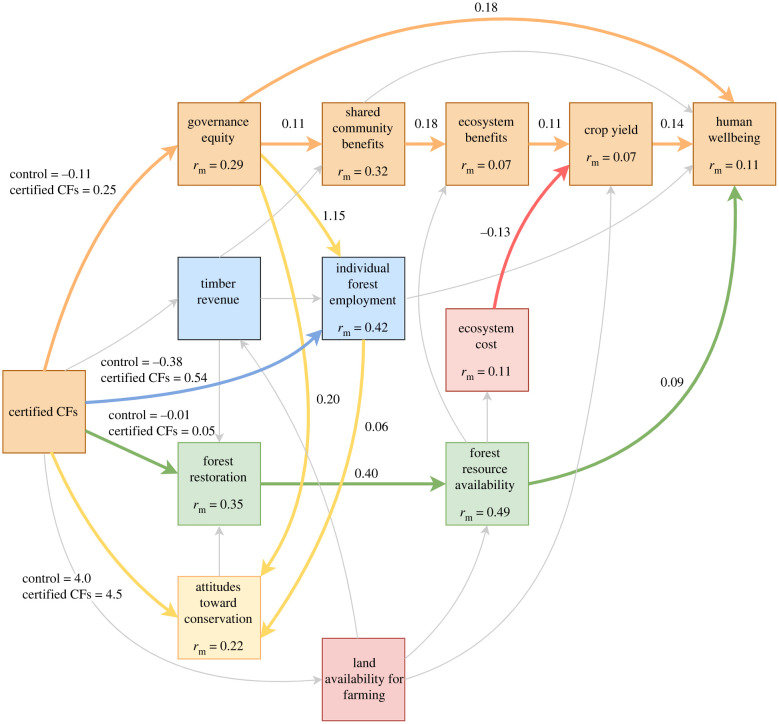
Conceptual model showing theoretical win-win pathways and locally perceived pathways. Colours highlight different pathways; orange = governance equity pathway, blue = financial pathway, green = conservation pathway; yellow = social interaction pathways, red = trade-off pathways. Grey arrows show paths removed from the simplified final structural equation model. Numbers show standardized path estimates for continuous variables and model estimates for binary variables for retained paths. Rm = marginal *r*^2^. (Online version in colour.)

We identified five main pathways linking governance of certified CFs to human wellbeing and forest restoration (figure 4). First, an equitable governance pathway (orange, figure 4), indicating that certified CFs were hypothesized to improve equitable governance, which would in turn positively impact human wellbeing. The most stated wish by community actors was that CF benefits be fairly distributed, e.g.

One of our expectations was to make sure the community forest benefits all villagers. – Community member, village 5.

Testing of our quantitative questionnaire showed that certified CFs positively impacted equitable governance (control estimate = −0.11, certified CF estimate = 0.25), with mean scores of all equitable governance indicators (participation, trust, satisfaction) higher in villages with certified CFs than control villages. In turn, equitable governance positively impacted human wellbeing both directly (standardized estimate = 0.18) and indirectly (figure 3). The indirect pathway occurred because equitable governance predicted perceived improvements in shared community development benefits (standardized estimate = 0.11). Timber revenue in certified CFs was most commonly spent on improvements to (i) healthcare facilities e.g. solar lights, (ii) education e.g. school meals and (iii) water infrastructure e.g. for drinking and irrigation. Irrigation was particularly important because the dominant livelihood activity was small-scale cultivated agriculture. These infrastructure improvements had a positive impact on ecosystem service benefits represented by perceived soil fertility, firewood and water access (standardized estimate = 0.18). Ecosystem benefits positively impacted crop yield (standardized estimate = 0.11), which in turn had a positive impact on human wellbeing (standardized estimate = 0.14; figure 3).

Second, a financial benefits pathway (blue, figure 4), whereby certified CFs are hypothesized to increase timber revenue, providing paid forest employment opportunities and direct financial benefits for individual community members, as well as contributing to community development projects. However, in our case study example the financial pathway did not impact forest restoration or wellbeing. Certified CFs did not impact timber revenue and although certified CFs impacted forest employment (control estimate = −0.38, certified CF estimate = 0.54), forest employment did not impact wellbeing. Qualitative data corroborated these quantitative findings, with concerns raised in four of the six certified CF villages consulted during exploratory focus groups, for example:

We have a lot of forest here and we work to manage it. But we have a limited number of customers and so the villagers here have not yet felt the actual benefit of this forest. But if we could get many customers to buy our timber then every member of this village could realize the importance of managing the forest - Village Natural Resource Committee Secretary, village 11.

However poor economic performance was not the case in all villages. Annual timber revenue was highly variable between certified CFs (mean of 12.75 USD per person; range 1.17–80). The size of certified CFs was also highly variable (mean of 14 320 ha; range 920–64 550), with larger CFs generating more revenue. Some differentiation in timber revenue spending was observed between villages; the highest timber producing village able to undertake additional, larger-scale and more diverse community development projects, including (i) building new village government offices, (ii) installing primary school sanitation facilities, (iii) improved village healthcare provision targeting facilities for pregnant women and disabled patients and health insurance for village natural resource committee members, (iv) building a village-run guesthouse, (v) payment for forest patrols, planning meetings and patrol equipment, (vi) payment for professional forestry and governance training services from the supporting NGO MCDI and the district government forest office. An FSC-certified sawmill factory run by a sustainable timber production company called Sound and Fair had also been established in this village in 2017 to further up-scale timber production and revenue generation, providing additional employment opportunities.

Third, a conservation pathway (green, figure 4) showed that certified CFs positively impacted forest restoration (control estimate = −0.01, certified CF estimate = 0.05)), which in turn positively impacted perceived availability of forest resources (standardized estimate = 0.40) and availability of forest resources had a positive impact on human wellbeing (standardized estimate = 0.09). An indirect conservation pathway was also hypothesized, whereby availability of forest resources would impact provision of ecosystem benefits, with a knock-on positive impact on crop yield. However, forest resource availability did not predict ecosystem benefits in our case study.

Fourth, positive interaction pathways were hypothesized from social to conservation outcomes (yellow, figure 4) with positive social outcomes driving improved attitudes towards conservation and forest restoration. In our case study, both governance equity (standardized estimate = 0.20) and forest employment (standardized estimate = 0.06) had a positive impact on attitudes toward conservation. However quantitative analyses showed that attitudes towards conservation did not have an impact on forest restoration. Likewise, timber revenue did not predict forest restoration, despite timber sales being used in part to fund forest management:

We are funding our own forest management activities. We pay even from our own village basket for meetings and patrols - Village Natural Resource Committee member, village 2.

Fifth, trade-off pathways were hypothesized between conservation and agriculture (red, figure 4). Focus groups in communities identified the widespread perception that crop damage from wild animals was a serious problem and that this was worse near forests (ecosystem costs). In our case study, ecosystem costs had a negative impact on reported crop yield (standardized estimate = −0.13). However, ecosystem costs was not predicted by our measure of forest resource availability despite anecdotal evidence from key informant interviews associating healthy forests with an increase in crop damage. A further trade-off pathway was identified in three of the villages where expansion of PAs was causing concerns about land shortages for farming:

The government extended the national forest reserve boundary and so we have been left with a small area for farming. That land, it was very fertile, it was supporting us to have high production and we had a lot of food surplus. But now we have little food because we harvest very little – Community member, village 1.

Larger certified CFs were also suggested by MCDI to have more economic potential for timber revenue, creating a trade-off between land uses. However, testing of our conceptual model did not show an impact of certified CFs on perceived land availability or knock-on impacts of perceived land availability for farming on crop yield or timber revenue.

## 4. Discussion

We found evidence of both win-win and trade-off pathways from certified CF governance to forest recovery and human wellbeing. Certified CFs positively impacted (i) equitable governance and (ii) forest recovery and both pathways positively impacted wellbeing, supporting win-win assumptions that positive social and conservation outcomes can occur together and are causally linked. However, additional hypothesized win-win pathways of (iii) financial benefits from certified CFs and (iv) improved attitudes towards conservation did not impact either wellbeing or forest restoration, suggesting that the importance of these pathways was limited in our case study. The limitation of the economic pathway linking forest governance to wellbeing may be due to the FSC timber market being at an early stage of development and operating sub-optimally. While some villages were able to generate significant FSC timber revenue, which was used to deliver integrated programmes to improve human wellbeing, concerns were raised by other villages about the challenges of accessing timber markets. Nevertheless, these findings agree with other research suggesting that equitable governance can be a more important driver of successful conservation than financial incentives [11]. Finally, trade-off pathways between conservation and agriculture, and the importance of agriculture for wellbeing provide contrary evidence that conservation and wellbeing objectives may be competing. These findings show that no simple solution exists for meeting multiple objectives in PA governance.

By disaggregating the impacts of PA governance into multiple pathways it is possible to identify which aspects of an intervention are performing well and which aspects are failing. In our case study, the governance equity component of certified CFs had a positive impact, in contrast to other CF programmes in Tanzania [41]. However, forest employment did not have an impact on wellbeing. This suggests that the hypothesized financial benefits aspect of certified CFs was underperforming, like other CF programmes in Tanzania [42] and market-based approaches across the tropics more generally [43].

By comparing quantitative and qualitative findings it is possible to explore how variation in contextual factors can lead to alternative outcomes. Not all CFs showed poor financial performance. The village that  established the largest CF, was also generating the most timber revenue, which was spent on diverse community projects, including additional income generation schemes such as a village-run guesthouse. The economic potential of this village had also attracted the establishment of an FSC-certified sawmill, in contrast to other villages which struggled to attract timber buyers. This more economically successful village, contrary to the general trend identified by modelling analyses, suggests that improved financial performance may require villages to dedicate significant land area to CFs and that strategies are needed to improve engagement with timber markets.

The positive interaction pathways suggest the potential for virtuous cycles to occur over time [44], where positive social impacts caused by certified CFs would improve attitudes toward conservation, leading to forest recovery, which would drive an upwards spiral of continuous improvements in social and ecological outcomes. However, evidence to support the presence of social interaction pathways was limited, with the generally poor financial performance of CFs constraining community investment in forest management. Testing of this hypothesized virtuous cycle would require time-series data to show change over time and evaluation of feedback mechanisms through more complex, non-recursive structural equation models. For example, testing forest governance impacts wellbeing and then reciprocally how changes in wellbeing might impact forest governance. Exploration of virtuous cycles represents a future research direction with potential to identify win-win pathways that would amplify the benefits of longer-term interventions.

The trade-off pathways, whereby forests provide both ecosystem benefits and costs for agriculture and potential agricultural land-shortages caused by expansion of PAs, suggest that the study landscape represents a microcosm of global challenges to reconcile forest conservation with rural development objectives [1,5]. We did not find significant evidence of land-shortage trade-offs. However looking to the future, as waves of forest degradation and land-use change penetrate deeper into rural areas of low income countries [45], these trade-offs between agriculture and conservation will likely become more acute. The agriculture–conservation trade-off represents a potential crossroads in land-use decision-making that will determine whether the landscape follows the same trajectory of current global trends, prioritizing agricultural expansion and forest degradation [5]. Alternatively, theory on transformative change suggests that to tip the system from one state (e.g. agricultural expansion) into another (e.g. sustainable use) requires disruption of the dominant drivers on the system [46]. Our conceptual model identifies key points in the system which could be leveraged to promote sustainable land-use choices. Specifically, mitigation strategies would be needed to minimize negative impacts of wild animals on crop production and efficient land-use planning to minimize land shortages, particularly for marginalized groups. Simultaneously, pathways of equitable governance, financial benefits and availability of forest resources, which all positively impacted wellbeing would need to be optimized to offset costs of foregone agricultural activity.

Our research design sort to embrace a complex systems perspective. However, several simplifications were necessary to aid interpretation. To support a statistical comparison, we employed a binary distinction between the governance approaches of certified CFs and control villages. However, we recognize that within this overarching governance grouping, varying governance arrangements exist. To move beyond a coarse binary description of governance approaches, we employed qualitative methods to highlight outlying cases that contrasted with the normative quantitative trends reported. However further exploration of the within-group variation in governance approach would be possible through more in-depth case study research [10]. Both human wellbeing and equitable governance are multidimensional concepts. However, these concepts were both consolidated into single indexes for the purposes of this study as a trade-off between complexity and simplicity to limit the number of pathways in the overall conceptual model to aid interpretation of dominant trends. This aggregation approach limits understanding of which specific dimensions of wellbeing and governance are being impacted by the intervention. Disaggregation of impacts between wellbeing domains for this dataset was undertaken in a separate study, which found that certified CFs positively impacted health, security and freedom domains of wellbeing, but not material wellbeing or social relations [47]. To strengthen causal inference in the research design a difference-in-differences approach might be employed to assess change between treatment and control groups through time. However, given the inductive selection of wellbeing indicators, such longitudinal data were not available for this study. Finally, although we found evidence that certified CFs positively impacted forest restoration, equitable governance and attitudes toward conservation, we were not able to confirm the intermediary mechanism linked certified CFs to improved forest restoration. This represents an important area of future research.

Our novel methodology illustrates the utility of a mixed methods approach for developing and testing theory of complex systems, with quantitative analyses showing overall trends, while qualitative analyses identifying alternative pathways missed by normative analyses. The integration of multiple actor perspectives provided a more comprehensive and contextualized understanding of pathways that balanced assumed positive and negative impacts of forest governance on people and forest recovery. By integrating views of actors from the global south our methodology makes progress in operationalizing calls for a pluralistic perspective of conservation challenges [48], to improve the equity of both the research process to conceptualizing challenges and design of effective solutions. As the role of PAs continues to diversify, methodological innovation is needed to understand how complex, positively and negatively interacting pathways can be navigated to promote forest recovery and human wellbeing. Through the combination of qualitative and quantitative methods, it is possible to move beyond a simplistic understanding of intervention performance, toward more in-depth understanding of what works, where and why.

## Data Availability

A text file of data and an r file of code is provided as supplementary material [49].
